# Pulsed radiofrequency or anterior neurectomy for anterior cutaneous nerve entrapment syndrome (ACNES) (the PULSE trial): study protocol of a randomized controlled trial

**DOI:** 10.1186/s13063-017-2110-5

**Published:** 2017-08-02

**Authors:** Robbert C. Maatman, Monique A. H. Steegers, Oliver B. A. Boelens, Toine C. Lim, Hans J. van den Berg, Sandra A. S. van den Heuvel, Marc R. M. Scheltinga, Rudi M. H. Roumen

**Affiliations:** 1 Department of General Surgery, Máxima Medical Centre, PO Box 7777 5500 MB, Veldhoven/Eindhoven, The Netherlands; 2 SolviMáx, Center of Expertise for ACNES, Center of Excellence for Abdominal Wall and Groin Pain, Máxima Medical Centre, Eindhoven, The Netherlands; 3 Department of Anaesthesiology and Pain and Palliative Care, Radboud University Medical Centre, Nijmegen, The Netherlands; 4 Department of Surgery, Maasziekenhuis Pantein, Boxmeer, The Netherlands; 5 Department of Anaesthesiology and Pain and Palliative Care, Máxima Medical Centre, Veldhoven, The Netherlands

**Keywords:** Abdominal pain, Randomized controlled trial, Chronic pain, Pain management, Surgical procedures, Operative, Pulsed radiofrequency treatment

## Abstract

**Background:**

Some patients with chronic abdominal pain suffer from an anterior cutaneous nerve entrapment syndrome (ACNES). This somewhat illusive syndrome is thought to be caused by the entrapment of end branches of the intercostal nerves residing in the abdominal wall. If ACNES is suspected, a local injection of an anesthetic agent may offer relief. If pain is recurrent following multiple-injection therapy, an anterior neurectomy entailing removal of the entrapped nerve endings may be considered. After 1 year, a 70% success rate has been reported. Research on minimally invasive alternative treatments is scarce. Pulsed radiofrequency (PRF) treatment is a relatively new treatment for chronic pain syndromes. An electromagnetic field is applied around the nerve in the hope of leading to pain relief. This randomized controlled trial compares the effect of PRF treatment and neurectomy in patients with ACNES.

**Methods:**

Adult ACNES patients having short-lived success following injections are randomized to PRF or neurectomy. At the 8-week follow-up visit, unsuccessful PRF patients are allowed to cross over to a neurectomy. Primary outcome is pain relief after either therapy. Secondary outcomes include patient satisfaction, quality of life, use of analgesics and unanticipated adverse events. The study is terminated 6 months after receiving the final procedure.

**Discussion:**

Since academic literature on minimally invasive techniques is lacking, well-designed trials are needed to optimize results of treatment for ACNES. This is the first large, randomized controlled, proof-of-concept trial comparing two therapy techniques in ACNES patients. The first patient was included in October 2015. The expected trial deadline is December 2017. If effective, PRF may be incorporated into the ACNES treatment algorithm, thus minimizing the number of patients requiring surgery.

**Trial registration:**

Nederlands Trial Register (Dutch Trial Register), NTR5131 (http://www.trialregister.nl/trialreg/admin/rctview.asp?TC=5131). Registered on 15 April 2015.

**Electronic supplementary material:**

The online version of this article (doi:10.1186/s13063-017-2110-5) contains supplementary material, which is available to authorized users.

## Background

Chronic abdominal pain originating in the abdominal wall is termed chronic abdominal wall pain (CAWP). A CAWP syndrome may be caused by the anterior cutaneous nerve entrapment syndrome (ACNES). At present, ACNES is still often neglected as a possible cause of abdominal pain and discomfort and a frequently overlooked diagnosis [1, 2]. An exact pathophysiological explanation of the syndrome is currently lacking but may be related to alterations in abdominal wall neuroanatomy.

The abdominal wall is sensory innervated by anterior and lateral cutaneous branches of the anterior rami of the thoracic intercostal nerves (7th to 12th) [3]. In ACNES, normal function of one or more cutaneous branches of the thoracic intercostal nerves is disturbed by a hitherto unidentified event [3]. If ACNES is suspected, current treatment options include analgesics, subfascial injections of a local anesthetic (whether or not combined with a long-acting corticosteroid), transcutaneous electrical nerve stimulation (TENS) and surgical interventions such as anterior and posterior neurectomy. Injection therapy is effective in one third of patients in the long term [4]. A neurectomy is usually considered in the remaining two thirds with a reported 70% success rate [5].

Although neurectomy is effective in most patients, a less-invasive procedure may be of potential benefit. Pulsed radiofrequency (PRF) is a relatively minimally invasive treatment that was initially designed as a less destructive approach when compared to radiofrequency (RF) therapy. Using the intermittent administration of high-frequency currents, tissue temperatures do not exceed 42 °C, thus preventing neuronal damage [6, 7]. A number of clinical studies have shown potential as levels of chronic pain in a variety of pain syndromes were significantly reduced [8, 9]. Evidence regarding the use of PRF in ACNES is limited to two case reports on PRF treatment of the dorsal root ganglion (DRG) resulting in pain reduction and improved quality of life [10, 11].

The objective of the present paper is to discuss a randomized trial comparing PRF with neurectomy as treatment options in ACNES. Neurectomy is nowadays considered the “gold standard” treatment but less invasive methods may potentially be of benefit.

## Methods

### Trial design

This prospective, multicentre, non-blinded, proof-of-concept, randomized controlled trial (with a one-way optional crossover at 8 weeks) is performed in the SolviMáx Center of Expertise for ACNES and Center of Excellence for Abdominal Wall and Groin Pain and Maasziekenhuis Pantein, Boxmeer, The Netherlands. SolviMáx is a subdivision of the Surgical Department of Máxima Medical Center (MMC), a teaching hospital situated in the southern part of The Netherlands. The Dutch Ministry of Health, Welfare and Sport has certified SolviMáx as a Center of Expertise for ACNES treatment. The trial will be based on a clinical proof-of-concept design in order to investigate a potential difference in pain relief following either PRF treatment or an anterior neurectomy in ACNES. Furthermore, it is designed to attain more knowledge on the use of PRF on peripheral nerves and to detect possible side effects.

The present trial follows the guidelines of the Declaration of Helsinki (version 19 October, 2013). The protocol (protocol number NL53171.015.15) is approved by the Medical Ethics Committee of MMC. The study protocol (version 1) is registered at www.trialregister.nl (NTR registration number: 5131, date of registration 15 April 2015). The present paper is written according to the Standard Protocol Items: Recommendations for Interventional Trials (SPIRIT) 2013 Statement for reporting a clinical trial protocol [12]. The SPIRIT Checklist is provided as Additional file 1.

### Participants

Patient enrollment started in October 2015. Patients are identified at the two hospital facilities. Criteria for the diagnosis ACNES are (1) a constant site of tenderness that is superficially located covering a fingertip-sized point of maximal pain at the lateral border of the rectus abdominis muscle, (2) a somewhat larger area of altered skin sensation covering this tender point and (3) observing that tenderness increases by abdominal muscle tensing using Carnett’s test [13, 14]. Only adult patients (over 18 years old) diagnosed with unilateral ACNES and having temporary success from an injection regimen will be invited for participation. In ACNES, a treatment regimen consists of local abdominal wall infiltration using 5–10 mL lidocaine as described in our earlier studies [4, 15]. “Temporary” is defined as having more than 50% pain reduction for at least 1 week after such a local infiltration although symptoms recur afterwards (refractory ACNES). Sixty-six patients (male or female) will be enrolled in the trial. Patients are not eligible if pain is caused by surgical scar-related pain syndromes (i.e., point of maximum pain is located at the site of a surgical scar) or due to recent intra-abdominal pathology. Presence of other chronic pain syndromes including fibromyalgia, dystrophy, chronic low back pain, impaired communication, a previous spinal surgical procedure at or between vertebral levels T7 and L1 are also exclusion criteria. A full list of inclusion and exclusion criteria is given in Table 1. Once eligibility is determined, patients are counseled on the specifics of the study and are given a number of days prior to providing consent.

**Table 1:** Subject inclusion and exclusion criteria

Inclusion criteria	Exclusion criteria
• Patient is diagnosed with unilateral anterior cutaneous nerve entrapment syndrome (ACNES)	• Patient has surgical scar-related pain syndromes
• Eligible for neurectomy (i.e., having temporarily success using injection therapy)	• Patient has recent intra-abdominal pathology
• Patient >18 years old	• Patient has other chronic pain syndromes (such as fibromyalgia, dystrophy, chronic low back pain)
• Patient is able to provide written informed consent	• Patient has other neuropathic diseases
• Patient is willing to participate in the follow-up schedule and protocol	• Patient has impaired communication
	• Patient has participated in another clinical investigation within 30 days
	• Patient has had a spinal surgical procedure at or between vertebral levels T7 and L1
	• Patient has been diagnosed with cancer in the past 2 years, except for skin malignancies
	• Female patient of childbearing potential is pregnant/nursing or plans to become pregnant during the course of the trial
	• Significant anatomic deformity (either congenital or acquired)
	• Language barrier
	• Allergy to local anesthetics

### Interventions

#### PRF arm

**Fig. 1:**
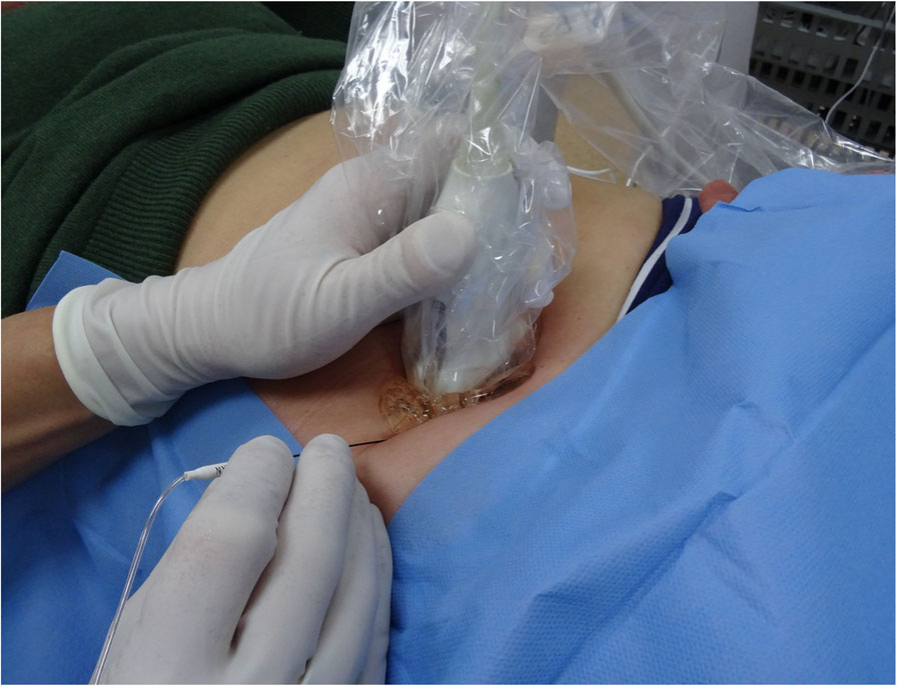
Ultrasound-guided placing of the pulsed radiofrequency (PRF) cannula at the tender point

Patients will be randomized to one arm of treatment, either PRF or an anterior neurectomy. Patients assigned to the PRF arm will visit departments of pain management of both hospitals for PRF treatment. While supine, a maximal point of pain is determined by asking and by a physical examination. In ACNES, there is characteristically a small (<2-cm^2^) constant site of anterior abdominal tenderness. Following marking, the skin is prepped with betadine and draped. Ultrasound (US) is used to locate the anterior fascia of the rectus abdominis muscle. The skin is locally anesthetized using 1% lidocaine. A straight, sharp RF cannula (SMK Pole needle 54 mm with 5-mm active tip, Cotop International BV, Amsterdam, The Netherlands) is inserted at an approximately 45° angle through the skin (Fig. 1). The tip of the cannula is then positioned between the anterior and posterior fascia of the rectus abdominis muscle. Electrical impedance is checked confirming a normal, closed electrical circuit. Subsequently, the sensation testing mode (50 Hz, 0.3–0.5 V) is started. As the nerve is often not visible using US, this step is crucial for nerve localization. Sensations such as paresthesia, numbness or prickly sensations should occur at less than 0.5 V if the needle’s position is correct [16]. The cannula is subsequently connected to the PRF Generator (G4, Cosman Medical, MA, USA) using the following settings: 45 V, <42 °C, 20 msec and 2 Hz. Treatment is applied for 6 min.

#### Neurectomy arm

**Fig. 2:**
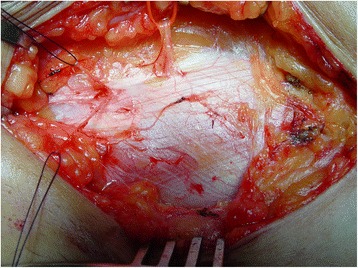
Intraoperative view of a neurovascular bundle (loop) and a nearby branch (loop) perforating the fascial foramina of the anterior sheath of the abdominal rectus muscle

Patients assigned to the neurectomy arm will be operated on in a daycare setting. The area of maximal pain is identified and marked. Once general anesthesia is administered, the anterior sheath of the rectus abdominal muscle is exposed via a ±5-cm transverse skin incision. The neurovascular bundle penetrating into the subcutaneous fat through the pre-existent fascial foramen is identified (Fig. 2). The fascia is widened and the bundle and all its branches within a 5-cm radius are ligated and removed. Accompanying vascular structures are also ligated or coagulated. The sheath as well as the remainder of the wound are closed in layers using absorbable suturing material.

#### Medication

“Escape” medication as currently used for pain reduction is allowed to be continued during the entire study period. In daily clinical practice, patients who underwent a neurectomy are always allowed to take analgesics in the postoperative period. Both groups are allowed to take medications according to their own need whereas quantities are tabulated.

### Outcomes

**Fig. 3:**
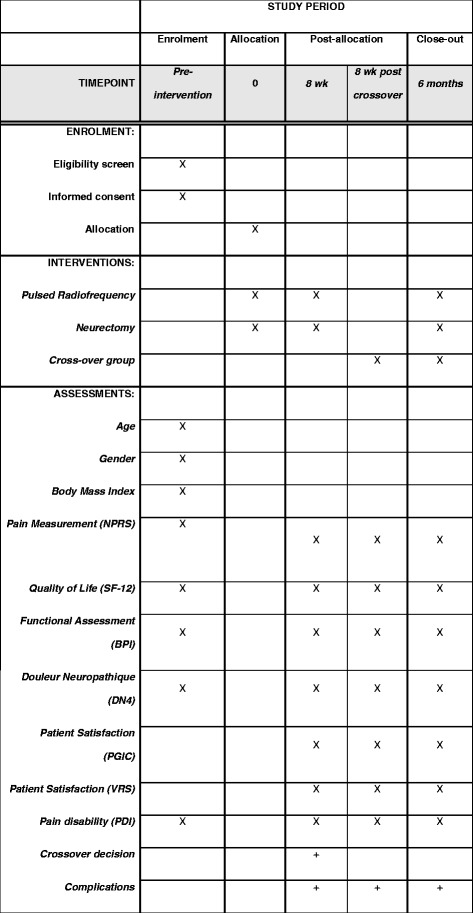
Content for the schedule of enrollment, interventions and assessments, according to the Standard Protocol Items: Recommendations for Interventional Trials (SPIRIT) Statement [12]

The primary objective is to compare the effect of PRF with a neurectomy in terms of pain relief at the 8-week follow-up. The outcome is measured using the Numeric Pain Rating Scale (NPRS, 0 = no pain and 10 = excruciating). Pain is measured at t_0,_ before intervention and 8 weeks after allowing the determination of short-term efficacy (t_1_) as shown in the schedule of enrollment, interventions and assessments, according to the SPIRIT Statement (Fig. 3). Long-term efficacy is measured at 6-month follow-up (t_2_). Success is defined as more than 50% NPRS pain reduction following intervention.

Secondary outcomes include the effect of PRF or neurectomy on quality of life, disability, neuropathic characteristics, medication usage and satisfaction. The Short Form Health Survey-12 questionnaire (SF-12) is used for measuring quality of life and interference from pain [17]. Patient disability will be measured with the Pain Disability Index (PDI) and the Brief Pain Inventory (BPI) [18, 19]. The Douleur Neuropathique (DN4) is used in order to discriminate between neuropathic and non-neuropathic pain [20]. Medication usage prior to, and after, treatment will be recorded using World Health Organization (WHO) pain steps. Patient satisfaction is recorded using the Patient Global Impression of Change (PGIC, 1 very much worse to 7 very much improved) and Verbal Rating Scales methodology (VRS, 1 = “I am very satisfied” and 5 = “Pain is worse after treatment”) [4, 21].

All adverse events (AEs) reported spontaneously by the patient, or observed by the investigators or their staff, will be recorded.

### Data handling

The investigators and co-investigators will make every reasonable effort to protect the confidentiality of the patients participating in the trial. Patients will not be identified by name, social security number, address, telephone number, or any other direct personal identifier. A unique identification code will be assigned to each patient participating in this trial. Information about the code will be kept in a secure location. Data storage will reside at the coordinating site, MMC, in locked offices. Sites will retain collected data for a minimum of 15 years. All electronic data will be password-protected on computers stored in locked offices. Access to patient information will be limited to trial personnel only.

### Sample size

Sample size estimation is based on a “responder” analysis, as recommended by the Initiative on Methods, Measurement and Pain Assessment in Clinical Trials (IMMPACT) Group recommendations [21]. Effect sizes were estimated on the basis of published academic literature. Neurectomy is considered successful in 70% [13, 22–25]. Regarding the potential efficacy of PRF treatment, a mean effect size was estimated at 30%. There is no available academic literature apart from two case reports on the use of PRF in ACNES patients. The targeted effect size is a difference in proportion responding to treatment (neurectomy versus PRF) of 40%; i.e., 70% responding in neurectomy the group versus 30% in the PRF group. Using G*Power 3.1.7 software, 80% power and a two-tailed alpha of 5%, 58 participants are needed to demonstrate a potential effect of either type of treatment on pain relief. With an allowance for attrition of 10% in both arms at 6-month follow-up, we will aim to recruit a total of 66 patients.

### Randomization

After enrollment and completing the baseline questionnaires, patients are randomly assigned (1:1 – PRF:neurectomy) to one of two treatment groups following a computer-generated list of random numbers by blocks of 8. Randomization is stratified by treatment site location (Boxmeer or Veldhoven). The allocation sequence is concealed from the enrolling researcher and assessing participants in sequentially numbered, opaque and sealed envelopes, prepared by a secretary with no involvement in the trial. One central coordinating investigator (RM) is responsible for enrolling patients and is the only investigator allowed to inform the independent secretary of newly enrolled patients.

### Blinding

Blinding of patients, surgeons and pain specialists is not possible due to the characteristics of both treatments (minimally invasive treatment without general anesthesia versus invasive treatment with the use of general anesthesia).

### Statistical methods

All analyses are performed using the Statistical Package for the Social Sciences (SPSS) version 21 for Windows. Categorical variables are described as frequencies. Continuous data are tested for normality and are presented as means with standard deviation (±SD) or median values (range) as appropriate. The primary outcome measure is pain relief using the NPRS as compared to the preintervention pain levels (t_0_). Data of the PRF and neurectomy groups will be compared using the Student’s *t* test or the Wilcoxon signed-rank test, as appropriate. Secondary outcomes will be compared between groups at various time points (baseline, 8-week follow-up and 6-month follow-up). They will be compared to preintervention values using Student’s *t* test or the Wilcoxon signed-rank test as appropriate. A *p* value < 0.05 is considered significant. Analysis of data will be done as randomized (the intention-to-treat analysis) and secondary “as-treated.”

### Recruitment

Potential patients are identified by physicians who are working in abdominal pain clinics at the SolviMáx or the Maasziekenhuis Pantein and are screened for eligibility. Patients are then informed on the purpose, nature and duration of the trial. Following consultation, potential participants are allowed 14 days for consideration. If a patient subsequently consents, they are then randomized by the principal investigator. Patients are allowed to withdraw their consent at any given time during the study period.

## Discussion

Anterior cutaneous nerve entrapment syndrome (ACNES) is caused by the entrapment of end branches of the intercostal nerves that are residing in the abdominal wall. Patients suffer from severe abdominal pain that is often not recognized as most physicians are focused, when confronted with abdominal pain, on a visceral source of the pain [1, 2]. The diagnosis of ACNES is suggested by a specific combination of the patient’s history (chronic pain) and physical examination (circumscript pain localization, positive pinch, Carnett’s test and abnormal sensitivity) and the absence of objective abnormalities in either laboratory tests or imaging techniques searching for possible visceral causes [4]. Once patients are diagnosed with ACNES, a treatment regimen including tender-point injections is subsequently offered [4]. If the pain is recalcitrant, a neurectomy is considered. This treatment algorithm is successful in up to 90% of patients [5]. Research on minimally invasive treatment options is exceedingly scarce but may be explored as suggested [26–28]. Two case reports recently attracted attention to PRF treatment of the dorsal root ganglion (DRG) as a potential alternative approach in ACNES resulting in pain reduction and improvement of quality of life [10, 11]. The present study is the first randomized trial comparing PRF and neurectomy in ACNES patients. Results of this proof-of-concept trial may determine whether PRF offers an effective treatment option for ACNES.

PRF treatment was initially designed as a less destructive alternative to RF therapy. The technique is based on the intermittent administration of high-frequency current resulting in tissue temperatures below 42 °C, thus preventing neuronal damage [6, 7]. Initial studies were promising reporting significantly reduced levels of chronic pain in a variety of syndromes [8, 9]. However, evidence on its use on peripheral nerves is scarce. In the present era of evidence-based medicine, well-designed proof-of-concept trials are required prior to widespread introduction. PRF is an example of a popular treatment tool for several chronic pain conditions although the scientific evidence is rather limited [29–31]. The effect of PRF may depend on the type of pain syndrome. Reports on PRF in cervical radicular pain and lumbosacral radicular pain suggested major pain relief for more than 3 months, providing level 1B+ and 2C+ evidence, respectively [8, 9]. However, the use of PRF in other pain entities, such as lumbar zygapophyseal joint pain and trigeminal neuralgia, was found to be less effective than conventional RF [32, 33]. Therefore, there is a need for high-level evidence studies confirming the possible beneficial effects of PRF treatment in specified pain syndromes.

A potential limitation of the present study is its non-blinded design. However, blinding patients and/or physicians in the present design is practically impossible.

In conclusion, this randomized controlled, proof-of-concept trial will investigate the possible efficacy of PRF treatment as a minimally invasive treatment in ACNES patients. If effective, patients could benefit from its less invasive character whereby the need for surgery is minimized. High-level evidence on the use of PRF treatment on peripheral nerve pain syndromes will be increased. The first study results are expected towards the end of 2017 and will be communicated via a publication.

### Trial status

Period of patient recruitment.

## Additional file

Additional file 1: SPIRIT 2013 Checklist of the PULSE trial. SPIRIT 2013 Checklist: Recommended items to address in a clinical trial protocol. (DOCX 41 kb)

## Abbreviations

ACNES: Anterior Cutaneous Nerve Entrapment Syndrome
AE: Adverse event
BPI: Brief Pain Inventory
CAWP: Chronic Abdominal Wall Pain
DN4: Douleur Neuropathique 4
DRG: Dorsal root ganglion
FU: Follow-up
IMMPACT: Initiative on Methods, Measurement and Pain Assessment in Clinical Trials
MMC: Máxima Medical Center
NPRS: Numerical Pain Rating Scale
PDI: Pain Disability Index
PGIC: Patient Global Impression of Change
PRF: Pulsed radiofrequency
RF: Radiofrequency
SF-12: Short Form Health Survey-12 questionnaire
SPSS: Statistical Package for the Social Sciences
TENS: Transcutaneous Electrical Nerve Stimulation
VRS: Verbal Rating Scale
